# Addressing under-registration in Chagas disease mortality: insights from the SaMi-Trop and REDS cohorts

**DOI:** 10.1590/S1678-9946202567010

**Published:** 2025-02-07

**Authors:** Ana Luiza Bierrenbach, Claudia Di Lorenzo Oliveira, Nayara Dornela Quintino, Nayara Ragi Baldoni, Carlos Henrique Valente Moreira, Ariela Mota Ferreira, Lea Campos de Oliveira da Silva, Márcio Oikawa, Maria do Carmo Pereira Nunes, Clareci Silva Cardoso, Desirée Sant’Ana Haikal, Fabio de Rose Ghilardi, Thallyta Maria Vieira, Antonio Luiz Pinho Ribeiro, Ester Cerdeira Sabino

**Affiliations:** 1 Hospital Sírio-Libanês, São Paulo, São Paulo, Brazil; 2 Universidade Federal de São João del-Rei, Divinópolis, Minas Gerais, Brazil; 3 Universidade de Itaúna, Itaúna, Minas Gerais, Brazil; 4 Universidade de São Paulo, Faculdade de Medicina, Instituto de Medicina Tropical de São Paulo, São Paulo, São Paulo, Brazil; 5 University of California, Division of HIV, Infectious Diseases and Global Medicine, San Francisco, California, United States; 6 Zuckerberg San Francisco General Hospital, San Francisco, California, United States; 7 Universidade Estadual de Montes Claros, Montes Claros, Minas Gerais, Brazil; 8 Universidade Municipal de São Caetano do Sul, São Caetano do Sul, São Paulo, Brazil; 9 Universidade Federal de Minas Gerais, Belo Horizonte, Minas Gerais, Brazil

**Keywords:** Chagas disease, Mortality, Under-registration, SaMi-Trop cohort, REDS cohort, Burden of disease

## Abstract

Chagas disease (ChD) remains a significant public health concern in the Americas, with challenges to accurately assessing its mortality burden due to under-reporting and misclassification. This study aimed to analyze mortality patterns of two cohorts of individuals with ChD—one comprising asymptomatic individuals with positive serology (REDS) and another with patients showing Chagas cardiomyopathy (SaMi-Trop)—to propose a method for estimating the potential under-registration of Chagas-related deaths and to find the factors influencing the identification of ChD as the underlying cause of death. We carried out a retrospective analysis of mortality data from these cohorts together with data on the Brazilian Mortality Information System. Causes of death were classified according to ICD-10 codes, and an expert review was used to find possible Chagas-related deaths. Logistic regression was used to explore predictors of ChD identification considering demographic and clinical variables. Of 2,488 patients, 381 died, 28.9% attributed to ChD, predominantly chronic ChD with cardiac involvement (B57.2). Using our method, we estimated a 53.8% potential under-registration rate for possible Chagas deaths. Males were negatively associated with Chagas disease identification, with an odds ratio of 0.52 (95%CI 0.24–1.1). No other significant associations were found, and the overall significance of the model was low. Our findings provide a potential measurement of under-registration, indicating that it may be substantial. These results underscore the need for improved identification and accurate reporting on death certificates. Strengthening the quality of mortality data is essential to understand Chagas-related mortality and guide public health strategies to reduce its impact.

## INTRODUCTION

Chagas disease (ChD), caused by the parasite *Trypanosoma cruzi*, constitutes one of the most neglected diseases in the Americas, affecting millions of people (primarily in rural and impoverished areas)^1^. This chronic disease, which can remain asymptomatic for decades, can cause serious complications, such as Chagas cardiomyopathy and megaesophagus, resulting in significant morbidity and, in some cases, premature death^2,3^. Despite efforts to control disease transmission, ChD continues to be a leading cause of morbidity and mortality in the region^4-7^.

A key challenge in accurately assessing the ChD burden refers to the underreporting of deaths from the disease in mortality systems^8^. Death certificates, the primary source for determining cause of death, are often unable to accurately identify deaths related to ChD. Studies have shown significant misclassification and underreporting of Chagas-related deaths on these certificates^9,10^. This misclassification occurs because symptoms of ChD, such as heart failure and gastrointestinal issues, can resemble those of other illnesses, resulting in vague coding on death certificate^3^. Additionally, healthcare providers certifying the causes of death often use codes for intervening and immediate causes of death rather than the underlying cause itself because they may be unaware of the underlying cause or be unable to accurately fill out such certificates^11^. This practice can lead to the incorrect attribution of the immediate cause, such as heart failure or pneumonia, without identifying ChD as the underlying cause. Consequently, the disease burden undergoes a considerable underestimation, hindering a comprehensive understanding of its actual severity and its implications for public health.

ChD leads to various complications that can ultimately result in death, with differing mechanisms based on whether the cardiovascular or digestive system is affected. In chronic Chagas cardiomyopathy, progressive heart muscle damage can lead to heart failure, which may directly cause death. Heart failure can also lead to fluid retention in the lungs, resulting in pneumonia and sepsis. Severe arrhythmias, including ventricular tachycardia and fibrillation, can result in sudden cardiac death, whereas thromboembolic events increase the risk of stroke, pulmonary embolism, or other organ infarctions. In the digestive form, complications such as megaesophagus and megacolon stand out. Megaesophagus can lead to aspiration pneumonia and severe malnutrition, whereas megacolon can cause intestinal obstruction, volvulus, or perforation, culminating in sepsis.

Physicians responsible for certifying causes of death must understand that these mechanisms are crucial for correctly attributing causes of death. Additionally, they should be familiar with WHO logic on the sequence of events that lead to death. WHO guidelines define the underlying cause of death as the disease or injury that initiated the chain of events directly leading to death^12^. Understanding this logic is essential to accurate fill out death certificates and ensure that ChD is appropriately identified as the underlying cause of death even if other immediate causes or complications are also listed.

This study analyzes registered mortality data from two cohorts of patients living with ChD that were followed-up over several years to assess patterns of ChD-related mortality and explore the extent of potential under-registration and its influencing factors. This analysis aims to raise awareness of the need to improve the quality of mortality records for ChD, ensuring more accurate identification and reporting of Chagas-related deaths^13,14^.

## MATERIALS AND METHODS

This study aims to comprehensively analyze mortality causes in patients in the SaMi-Trop and REDS cohorts, focusing on identifying those related to ChD. Thus, the following objectives were established:

- Firstly, to establish and document the primary causes of mortality in patients in both cohorts, paying particular attention to those associated with ChD.
- Secondly, to quantify the extent of underreporting of ChD as a documented cause of death within the studied population.
- Lastly, to show the factors influencing the identification or non-identification of ChD as the underlying cause of death.

Regarding methodology, this study uses primary data from the SaMi-Trop and REDS cohorts and secondary data from the Brazilian Mortality Information System (SIM), which were matched by record linkage methods. Key components of the methodology of these studies include:

The SaMi-Trop cohort was established as a prospective study focusing on adult patients diagnosed with chronic ChD cardiomyopathy. Patients in this cohort were drawn from 21 municipalities in Northern Minas Gerais State, Brazil, and enrolled in the study from 2013 to 2014^15^. Recruitment was based on electrocardiogram results that were carried out in 2011–2012 by the Telehealth Network.

To be included in the SaMi-Trop cohort, participants had to (1) self-report Chagas disease; (2) show specific electrocardiogram abnormalities—such as Q wave patterns suggesting prior myocardial infarction, complete intraventricular block (right, left, or unspecified), frequent premature beats (supraventricular or ventricular), significant ST segment or T wave changes, atrial fibrillation, flutter, supraventricular tachycardia, other major arrhythmias, advanced atrioventricular conduction abnormalities, pacemaker use, significant QT prolongation (QT index >115%), or ventricular hypertrophy, and (3) be aged 19 years or older. Exclusion criteria included pregnancy, breastfeeding, or life-threatening conditions associated with a life expectancy of less than two years.

All participants meeting the aforementioned inclusion criteria were tested for *T. cruzi* antibodies using chemiluminescent microparticle immunoassay, the negative results of which were verified by two additional enzyme immunoassays with distinct antigens. The cohort was composed of individuals with a confirmed seropositive diagnosis. Additional details on the study design and recruitment process are available elsewhere.

Conversely, the REDS cohort is a retrospective study involving initially healthy blood donors who tested positive for *T. cruzi* antibodies during donations made from 1996 to 2002 in the Sao Paulo and Montes Claros cities, Brazil^16^. During this period, Brazilian blood donation criteria required donors to be aged 18–65 years, weigh at least 50 kg, and show good health. Temporary deferrals applied to individuals with recent fever or infection (seven days), vaccinations (up to four weeks, depending on type), travel to malaria-endemic areas (12 months), pregnancy, and breastfeeding. Permanent deferrals included selected transmissible diseases such as Chagas, hepatitis B/C, HIV, or syphilis, cancer (except non-melanoma skin cancer), or high-risk behaviors such as intravenous drug use. Screening for *T. cruzi* antibodies used three serological methods—ELISA, hemagglutination, and immunofluorescence. The blood units that reacted to any of these assays were discarded. Asymptomatic Chagas donors (confirmed positive by all three assays) were included in this study. Although the REDS cohort also included age-, sex-, and period-matched seronegative controls, only seropositive individuals were included in this analysis.

Using the available nominal data collected from the SaMi-Trop and REDS cohorts, which includes patients’ names, their mothers’ names, municipality of residence, and date of birth, the Brazilian Ministry of Health was asked to perform record linkage with SIM. This linkage methodology, which was developed and is routinely employed by the Ministry staff, configures a robust process that is widely applied in research and routine surveillance. The SIM database in this study represents the final version approved by the Ministry of Health, having undergone comprehensive quality checks at municipal, state and federal levels. These checks include efforts to resolve ill-defined causes and investigate garbage codes, although not all such cases are fully resolved^17,18^.

The SIM data are derived from standard death certificates, which follow the World Health Organization format. Block V of the death certificate, titled “Conditions and Causes of Death,” requires physicians to document the sequence of events leading to death in Part I, starting with the immediate cause on line (a) and listing antecedent causes on subsequent lines, with the underlying cause—the condition initiating the chain of events—recorded on the final available line. Part II captures contributory conditions indirectly part of the causal chain that may have influenced the outcome. Trained coders at the municipal or regional levels assign ICD-10 codes to the reported conditions and apply international rules to confirm the underlying cause of death^19^. Table 1 presents an excerpt from Block V of death certificates: “Conditions and Causes of Death” to illustrate its structure and content.

**Table 1:** Excerpt from death certificates: information from Block V – Conditions and causes of death.

Causes of death	Record only one diagnosis per line	Approximate time between the onset of the condition and death	ICD
**Part I**			
Disease or condition directly causing death.	a.		
**Preceding causes** Morbid conditions (if any) that gave rise to the cause registered above with the underlying cause listed last.	Due to or as a consequence of: b.		
	Due to or as a consequence of: c.		
	Due to or as a consequence of: d.		
**Part II**			
Other significant conditions contributing to death but outside the causal chain listed above.			
		

The linkage output information, stripped of nominal data, identified deaths up to December 2021, providing a maximum follow-up period of nine years for SaMi-Trop and of 10 years for REDS. Data on proximal and distal causes of death, as per Part 1 of death certificates, and the underlying causes of death were collected for each death in the cohorts, along with information on location of death occurrence.

In the linked database, the overall proportion of deaths for each cohort and their total population was calculated. For the combined cohorts, the proportion of deaths attributed to specific cause categories, including Chagas disease (ChD), cardiovascular, digestive, ill-defined, and other causes, was determined based on the ICD-10 codes for the underlying cause. Additionally, the occurrence of Chagas disease within the lines (a) to (d) listed on Part 1 of the death certificate was examined. The number of deaths for each ICD code was then separately described and the codes, grouped into broader categories. Then, the potential for misclassification or underidentification of Chagas disease within the cardiovascular, digestive, and ill-defined categories was evaluated. This evaluation was conducted with input from two physicians who specialize in Chagas disease and who are skilled in death coding. They identified specific ICD-10 codes within these categories as potentially representing Chagas-related deaths. Based on their expert assessment, these codes were categorized under “possible Chagas deaths” (Table 2), encompassing all deaths linked to these specific codes.

**Table 2:** Classification of Chagas disease-related deaths and associated conditions, including confirmed and possible deaths due to Chagas disease in specific cohorts of seropositive individuals.

Confirmed Chagas disease deaths	
B57	Chagas disease
B57.0	Acute Chagas disease with heart involvement
B57.1	Acute Chagas disease without heart involvement
B57.2	Chagas disease (chronic) with heart involvement
B57.3	Chagas disease (chronic) with digestive system involvement
B57.4	Chagas disease (chronic) with nervous system involvement
B57.5	Chagas disease (chronic) with other organ involvement
K23.1	Megaesophagus in Chagas disease
K93.1	Megacolon in Chagas disease
**Possible Chagas disease deaths**	
I42.0	Dilated cardiomyopathy
I42.9	Cardiomyopathy, unspecified
I44	Left bundle branch atrioventricular block (all categories)
I48	Atrial flutter and fibrillation
I46.1	Sudden cardiac death, so described
I49.9	Unspecified cardiac arrhythmia
I50	Cardiac failure (all categories)
I51.7	Cardiomegaly
I51.9	Other specified heart diseases
I63.4	Cerebral infarction due to embolism of cerebral arteries
I63.5	Cerebral infarction due to thrombosis of cerebral arteries
I63.9	Cerebral infarction, unspecified
I64	Stroke, not specified as hemorrhage or infarction
I67.8	Other specified cerebrovascular diseases
I67.9	Cerebrovascular disease, unspecified
I69.3	Sequelae of cerebral infarction
I69.4	Sequelae of stroke, not specified as hemorrhage or infarction
K22.0	Achalasia of cardia
K56.0	Paralytic ileus
K56.2	Volvulus
K56.4	Other obstruction of intestine
K56.6	Other and unspecified intestinal obstruction
K56.7	Ileus, unspecified
K59.3	Megacolon, not elsewhere classified
R13	Aphagia and dysphagia
R57.0*	Cardiogenic shock
R98*	Death, not attended
R99*	Other ill-defined and unspecified causes of mortality

*These are very unspecific ICD-10 codes. We believe their inclusion as possible Chagas deaths can be justifiable in the specific context of our cohorts, in which all patients are seropositive for Chagas disease and, in the SaMi-Trop cohort, known to have heart disease.

To estimate what was defined as the percentage of underregistration of Chagas-related deaths, which reflects a combination of potential misclassification or underidentification of Chagas disease, the total number of deaths classified as confirmed Chagas deaths (based on ICD-10 codes directly related to Chagas disease) was first identified. Deaths classified under the previously defined category “possible Chagas deaths,” comprising specific codes within cardiovascular, gastrointestinal, and ill-defined categories identified as potentially representing Chagas disease were then added to analysis. The percentage of underregistration was calculated as the proportion of “possible Chagas deaths” relative to the combined total of confirmed and possible Chagas deaths, expressed as: Underregistration (%) = (Possible Chagas deaths / (Confirmed Chagas deaths + Possible Chagas deaths)) × 100.

In the final part of the analysis, a nested case-control design within the cohorts was adopted and multiple logistic regressions were conducted to analyze the factors associated with possible Chagas classification. The outcome variable was defined as follows: deaths possibly attributable to Chagas disease but unrecognized as such were coded as 1 (possible Chagas deaths) and confirmed Chagas deaths were coded as 0. The following variables were included as potential predictors: gender; age group (17–59, 60–69, 70–79, and 80+ years); use of benznidazole; the New York Heart Association functional class (which evaluates cardiac function in four levels); use of a permanent pacemaker; place of death (hospital or non-hospital), and cohort (SaMi-Trop or REDS). Variables were selected using backward stepwise elimination, and interactions between variables were examined.

This cohort study was approved by the Institutional Research Ethics Committee, National Commission of Ethics in Research (CONEP) under protocol number 179.685/2012. Linkage between cohort and mortality data was conducted by the Ministry of Health, which ensured that researchers only had access to deidentified mortality data. This study was funded by the National Institutes of Health (NIH) under grants U109AI098461 and U01AI68383.

## RESULTS

**Figure 1:**
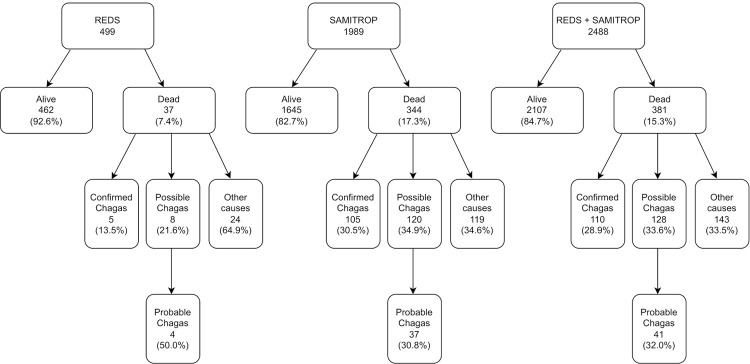
Classification of mortality outcomes in the REDS and SaMi-Trop cohorts: breakdown of Chagas disease attribution.

The breakdown of the patients’ vital status shows notable differences in the two cohorts (Figure 1). In the REDS cohort, 37 (7.4%) of 499 patients had died. In the SaMi-Trop cohort, 344 (17.3%) of 1,989 patients had died. Overall, of the combined total of 2,488 patients from both cohorts, 381 (15.3%) had died. Figure 1 also shows the final cause of death attribution for each cohort.

Table 3 shows the underlying causes of death for 381 patients from the REDS and SaMi-Trop cohorts as per the SIM and the classification by ICD-10 codes. The table provides the percentage of total deaths for each category and subcategory and the count and percentage of confirmed and possible ChD deaths. ChD constituted the underlying cause of 28.9% of all deaths, primarily attributed to chronic Chagas disease with cardiac involvement (B57.2). Notably, physicians registered Chagas disease codes exclusively as underlying causes of death, not appearing in any other lines (a-d) of patients’ death certificates. Cardiovascular, gastrointestinal, and ill-defined causes represented 30.2, 5.2, and 13.9% of deaths, respectively. Specific codes within these categories fell under the category ‘possible Chagas deaths.’ When combining confirmed Chagas deaths with those classified as possible, the total number of Chagas-related deaths increased to 238, representing a 53.8% under-registration.

**Table 3:** Underlying causes of mortality and Chagas disease attribution in deceased patients in the study population.

Underlying causes of death in SaMi-Trop and REDS patients according to the Brazilian Mortality Information System (SIM)	ICD-10 codes*	Groups n (%)	Categories n (%)	Confirmed Chagas deaths n	Possible Chagas deaths n
**Chagas**		110 (28.9)			
Acute Chagas disease	B57.0		1 (0.9)	1	
Chronic Chagas disease with cardiac involvement	B57.2		103 (93.6)	103	
Chronic Chagas disease with digestive system involvement	B57.3		5 (4.5)	5	
Chronic Chagas disease with nervous system involvement	B57.4		1 (0.9)	1	
**Cardiovascular**		115 (30.2)			
Hypertension	I10, I11.0, I11.9, I12, I13.2		12 (10.4)		
Coronary heart disease	I21.9, I24.8, I25.1, I25.8		22 (19.1)		
Mitral valve insufficiency	I34.0		1 (0.9)		6
Dilated cardiomyopathy	I42.0		6 (5.2)		3
Cardiomyopathy, unspecified	I42.9		3 (2.6)		1
Atrioventricular block, complete	I44.2		1 (0.9)		3
Atrial flutter and fibrillation	I48		3 (2.6)		3
Unspecified cardiac arrhythmia	I49.9		3 (2.6)		9
Congestive heart failure	I50.0		9 (7.8)		18
Heart failure, unspecified	I50.9		18 (15.7)		3
Heart disease, unspecified	I51.9		3 (2.6)		2
Intracerebral hemorrhage, unspecified	I61.9		3 (2.6)		14
Cerebral infarction, unspecified	I63.9		2 (1.7)		8
Stroke, not specified as hemorrhagic or ischemic	I64		14 (12.2)		
Other specified cerebrovascular diseases	I67.8		8 (7.0)		
Sequelae of stroke, not specified as hemorrhagic or ischemic	I69.4		6 (5.2)		
Aortic aneurysm and dissection	I71.0		1 (0.9)		
**Gastrointestinal**		20 (5.2)			
Achalasia of cardia	K22.0		1 (5.0)		1
Peptic ulcer, site unspecified, chronic or unspecified, with hemorrhage	K27.4		1 (5.0)		
Peptic ulcer, site unspecified, unspecified as acute or chronic, without hemorrhage or perforation	K27.9		1 (5.0)		
Diaphragmatic hernia without obstruction or gangrene	K44.9		1 (5.0)		
Noninfective gastroenteritis and colitis, unspecified	K52.9		1 (5.0)		
Acute vascular disorders of intestine	K55.0		1 (5.0)		
Paralytic ileus	K56.0		1 (5.0)		
Other obstructions of intestine	K56.4		1 (5.0)		
Other forms of intestinal obstruction, and unspecified	K56.6		6 (30.0)		1
Acute peritonitis	K65.0		1 (5.0)		1
Other specified forms of cirrhosis of liver	K74.6		1 (5.0)		6
Portal hypertension	K76.6		1 (5.0)		
Disease of gallbladder, unspecified	K82.9		1 (5.0)		
Acute pancreatitis	K85.9		1 (5.0)		
Hematemesis	K92.0		1 (5.0)		
**Ill defined**		53 (13.9)			
Respiratory arrest	R09.2		2 (3.8)		
Acute abdomen	R10.0		1 (1.9)		
Ascites	R18		1 (1.9)		4
Cardiogenic shock	R57.0		4 (7.5)		15
Death, not attended	R98		15 (28.3)		30
Other ill-defined and unspecified causes of mortality	R99		30 (56.6)		
**Others**	Multiple ICD-10 codes	83 (21.8)	83 (100.0)		
**Total**		**381 (100.0)**	**381 (100.00)**	**110**	**128**
**Under-registration (%)**					53.8

*ICD-10 = International Classification of Diseases – 10^th^ revision.

Table 4 shows the demographic and clinical characteristics of deceased patients, which this study categorized into Confirmed Chagas (n=110), Possible Chagas (n=128), and Other Causes (n=143). Across all groups, about one third of patients died before reaching age 60 years. This research observed significant gender differences, including a higher prevalence of women in the Possible Chagas group (62.5%) than in the Confirmed Chagas (46.4%) and Other Causes (47.6%) groups (p=0.017 for all groups, p=0.013 for Confirmed vs. Possible Chagas). Patients used pacemakers in significantly higher amounts in the Confirmed Chagas group (17.3%) than in the Possible Chagas (13.3%) and Other Causes (6.3%) (p=0.022) ones. However, a direct comparison between Confirmed and Possible Chagas groups was not significant (p=0.39).

**Table 4:** Demographic and clinical characteristics of deceased patients in the studied population.

Category	Value	1. Confirmed Chagas* (n=110)	2. Possible Chagas* (n=128)	3. Other causes* (n=143)	Total	p-value** 1 x 2 x 3	p-value*** 1 x 2
**Age group (years)**	17–59	40 (36.4%)	38 (29.7%)	42 (29.4%)	120 (31.5%)	0.20	0.65
	60–69	30 (27.3%)	37 (28.9%)	33 (23.1%)	100 (26.2%)		
	70–79	31 (28.2%)	38 (29.7%)	40 (28.0%)	109 (28.6%)		
	80+	9 (8.2%)	15 (11.7%)	28 (19.6%)	52 (13.6%)		
**Gender**	Female	51 (46.4%)	80 (62.5%)	68 (47.6%)	199 (52.2%)	0.017	0.013
	Male	59 (53.6%)	48 (37.5%)	75 (52.4%)	182 (47.8%)		
**Benznidazole use**	No	109 (99.1%)	124 (96.9%)	140 (97.9%)	373 (97.9%)	0.49	0.23
	Yes	1 (0.9%)	4 (3.1%)	3 (2.1%)	8 (2.1%)		
Functional class****	1	46 (42.2%)	56 (45.9%)	67 (46.9%)	169 (45.2%)	0.33	0.087
	2	16 (14.7%)	8 (6.6%)	16 (11.2%)	40 (10.7%)		
	3	47 (43.1%)	55 (45.1%)	57 (39.9%)	159 (42.5%)		
	4	0 (0.0%)	3 (2.5%)	3 (2.1%)	6 (1.6%)		
**Pacemaker use**	No	91 (82.7%)	111 (86.7%)	134 (93.7%)	336 (88.2%)	0.022	0.39
	Yes	19 (17.3%)	17 (13.3%)	9 (6.3%)	45 (11.8%)		
**Location of death**	Not in hospital	36 (32.7%)	37 (28.9%)	27 (18.9%)	100 (26.2%)	0.032	0.52
	In hospital	74 (67.3%)	91 (71.1%)	116 (81.1%)	281 (73.8%)		
**Cohort membership**	REDS	5 (4.5%)	8 (6.2%)	24 (16.8%)	37 (9.7%)	0.001	0.56
	SaMi-Trop	105 (95.5%)	120 (93.8%)	119 (83.2%)	344 (90.3%)		

*As defined in the methodology; **p-values were calculated using the chi-squared or Fisher’s exact tests for comparisons between all groups (1 × 2 × 3); ***p-values were calculated using the chi-squared or Fisher’s tests for comparisons between confirmed Chagas and possible Chagas groups (1 × 2); ****Functional Class data were missing for 11 patients.

The location of death significantly differed across all groups as most patients died in hospitals, especially in the Possible Chagas (71.1%) and Other Causes (81.1%) groups, when compared to the Confirmed Chagas one (67.3%) (p=0.032). This study found no significant differences by directly comparing the Confirmed and Possible Chagas groups (p=0.52). Cohort membership showed significant differences as most patients belonged to the SaMi-Trop cohort. The Confirmed Chagas (95.5%) and Possible Chagas (93.8%) groups had a higher proportion of SaMi-Trop members than the Other Causes group (83.2%) (p=0.001 for all groups). However, this research found no significant difference between Confirmed and Possible Chagas groups (p=0.56).

The logistic regression model for possible Chagas classification showed a significant association with sex (OR=0.51, 95%CI 0.3–0.87, p=0.013), indicating that men had a lower likelihood of being classified as possible Chagas deaths than women. None of the other variables included in the model showed significant associations. Our model showed a limited explanatory power, with a pseudo R^2^ value of 0.019, reflecting its restricted ability to address variance in Chagas classification based on the selected predictors. This analysis found no interactions between variables.

## DISCUSSION

Accurate identification of ChD as the underlying cause of death is critical for several reasons. First, it more realistically assesses the burden of ChD mortality, which is essential for informed resource allocation and public health planning. Second, its implications extend beyond the deceased by identify patterns and risk factors in affected populations. This, in turn, highlights groups at higher risk and informs strategies to improve diagnosis, treatment, and follow-up for individuals living with ChD, potentially reducing future deaths and improving patient outcomes. Finally, it better analyzes ChD epidemiology, a necessary step to develop effective prevention and control strategies.

The analysis of data from the SaMi-Trop and REDS cohorts provides valuable insights into the burden of ChD mortality and the challenges of accurately registering these deaths. According to the SIM data, ChD was registered as the underlying cause in 28.9% of deaths, predominantly attributed to chronic ChD with cardiac involvement (B57.2). Further examination suggests substantial under-registration, with rates estimated at 53.8% based on our classification method. This under-registration likely reflects systemic challenges in mortality registration and underscores the need for improved diagnostic and reporting practices.

Whereas it may be excessive to attribute all deaths categorized as ‘Stroke, not specified as hemorrhagic or ischemic’ to ChD, it is plausible that a substantial proportion of such deaths in a Chagas-infected population, particularly those with known cardiac involvement, could be due to ischemic strokes, likely of cardioembolic origin^9,20^. In the general population, approximately 80% of strokes are ischemic. However, it is reasonable to speculate that a considerable proportion of strokes in Chagas patients may be ischemic due to this cardiac complication^21^. Similarly, although directly attributing ileus to ChD fails to offer a definitive diagnosis, it is plausible to infer that a patient in a rural Chagas-endemic area of Minas Gerais with limited access to medical care might have experienced intestinal obstruction related to Chagas disease. The attending physician, with limited knowledge of the patient’s medical history or the pathophysiology of Chagas disease, might have recorded the cause of death as paralytic ileus^22,23^.

We also expected to find a high proportion of confirmed Chagas deaths, primarily as Chronic ChD with cardiac involvement. This expectation aligns with the epidemiology of Chagas disease, which indicates that cardiac involvement occurs more often than digestive manifestations, particularly in chronic cases, although the digestive form also carries a high mortality rate^24^. However, it is important to note that the method to select the underlying cause of death, as part of the SIM, might introduce a bias. This method tends to prioritize cardiac involvement over digestive manifestations. For instance, when mentioning Chagas cardiomyopathy (B57.2) and digestive manifestations (B57.3) on a death certificate, the underlying cause of death selector in the SIM will choose B57.2 as the underlying cause, regardless of its position on the certificate or the presence of other causes in the chain of causes indicating digestive manifestations as the leading cause of death. When this happens, B57.2 should be listed in Part II of the death certificate. Regional coders and health officials have previously raised concerns about this bias, suggesting the need to revise the selection algorithm, though further action is yet to be taken. It is noteworthy that our cohorts offered only one mention of Chagas disease on patients’ death certificates, which always served as the underlying cause.

The logistic regression models yielded low R^2^ values, indicating that the selected variables explain only a small proportion of the variance in the underreporting or under-identification of ChD as the cause of patient death. Despite this limitation, analysis showed a significant association with sex as male deaths were less likely to be classified as possibly due to ChD (OR=0.51, CI95% 0.3-0.87, p=0.013). This finding raises the question of whether male ChD patients may have higher mortality rates than female ones, potentially reflecting biological differences, disparities in healthcare access, or variations in disease presentation. Furthermore, women may die less frequently from ChD and more often from other causes, which could contribute to their lower probability of being classified as Chagas-related or as possible Chagas deaths^25^. These results underscore the need for gender-sensitive approaches to healthcare delivery and a deeper exploration of gender-specific factors influencing ChD diagnosis, care, and mortality reporting.

The higher mortality in the SaMi-Trop cohort agrees with its composition of patients with known Chagas cardiomyopathy, a condition associated with higher fatality risk. In contrast, the REDS cohort consisted of asymptomatic blood donors with Chagas serology, who were healthier at baseline. Although univariate analysis showed that 95.5% of confirmed Chagas deaths occurred in the SaMi-Trop cohort, and that the REDS cohort contributed proportionally more deaths attributed to other causes (16.8%), our multiple regression analysis found no significant differences in disease identification by cohort membership. This lack of difference likely reflects opposing factors influencing results. The SaMi-Trop cohort, drawn from regions with limited healthcare access, includes patients with cardiomyopathy—a condition more likely to be fatal and recognized as Chagas-related^15^. Conversely, the REDS cohort evaluated regions with better healthcare infrastructure and likely included fewer patients with cardiopathy, leading to a higher proportion of deaths classified under “other causes”^16^. Additionally, SaMi-Trop participants benefited from its strong efforts to communicate the nature of their condition and educate them on how to manage it. This likely enhanced patients’ understanding of their diagnosis and improved their ability to convey this information to healthcare providers, contributing to more accurate reporting of Chagas disease as the cause of death. These counterbalancing influences may explain the absence of significant differences in the multivariate analysis.

In our study population, the identification of Chagas disease (ChD) as a cause of death failed to significantly differ between deaths in hospitals and those outside them. This suggests that the quality of healthcare provided at the time of death may play a more critical role in accurate cause-of-death registry than its location. High-quality healthcare increases the likelihood of correctly diagnosing and registering underlying conditions such as ChD, whereas suboptimal care, particularly in resource-limited settings with limited expertise in ChD management, may hinder accurate attribution^26^. Additionally, hospital emergency services, in which many deaths occur, show unique challenges as patients often arrive in critical condition, limiting opportunities to obtain detailed medical histories^27^. The lack of a significant association between functional class and ChD identification further supports the idea that healthcare quality at the time of death may outweigh the influence of initial evaluations when patients entered their cohort. This finding agrees with the broader challenges of attributing causes of death in patients showing heart failure, as highlighted in the literature^28,29^.

A comparison of our findings with França *et al*.^10^ highlights the widespread issue of garbage codes concealing ChD deaths in Brazil. Reclassifying deaths originally under garbage codes as ChD-related could substantially increase the recorded mortality attributed to ChD, emphasizing the importance of the accurate classification of causes of death, especially in high-prevalence regions. It is worth noting that, for decades, the Brazilian Ministry of Health, in collaboration with state and municipal authorities, has tried to improve the quality of mortality data^14,30^. These efforts include investigations into poorly defined deaths and, more recently, the identification and correction of “garbage codes” by surveillance agents, which can potentially alter the underlying cause on the SIM.

This study has several limitations that must be acknowledged. First, its use of two cohorts—one comprising asymptomatic individuals with positive Chagas serology and another of patients with Chagas cardiomyopathy—introduces heterogeneity as their populations’ baseline health status, healthcare access, and disease progression significantly differ from each other. However, this study aimed to explore mortality patterns and the identification of ChD across diverse populations rather than directly comparing them. Second, the retrospective nature of this analysis and its reliance on record linkage with the SIM offer inherent challenges. The absence of a unique identifier in the database required probabilistic linkage methods, which, while robust, are less precise than direct linkage and could introduce errors in matching records. Furthermore, regional differences in healthcare quality, registry practices, and data completeness in the SIM—particularly in resource-limited settings with insufficient diagnostic expertise in ChD—may affect the accuracy of results and complicate their interpretation. Additionally, while our classification of possible Chagas deaths aimed to estimate potential under-registration, we are unable to state that all records classified as possible Chagas deaths were truly Chagas-related. Validating these classifications would require further investigation, and if some cases were Chagas-unrelated, this would reduce our under-registration estimate. Despite these limitations, this study provides valuable insights into the challenges of registering Chagas-related mortality and highlights the need for improved reporting practices.

## CONCLUSION

In conclusion, our study emphasizes the critical need for accurate methods to diagnose and record Chagas disease-related deaths, particularly in regions with a high disease burden. Improving the identification and recording of the underlying cause of death can enhance our understanding of the true burden of Chagas disease mortality and implement more effective strategies for its prevention and control.
